# Supplementary material for: Pinpointing Functionally Relevant miRNAs in Classical Hodgkin Lymphoma Pathogenesis
Source: Cancers (Basel). 2024 Mar 12;16(6):1126. doi: 10.3390/cancers16061126 (PMC10968648; doi:10.3390/cancers16061126)
Supplement: Supplementary file 1 [file cancers-16-01126-s001.zip › cancers-2894588-supplementary.pdf]

Supplementary Table S1. Human miRNA profiling studies of cHL

	HRS cells vs GC-B	cHL cell lines vs GC-B	cHL cell lines vs RLNs	cHL vs NHL cell lines
1. (1)			√ (RT-qPCR)	
*2. (2) **				√ (RT-qPCR)
3. (3)	√ (RT-qPCR)	√ (RT-qPCR)		
*4. (4) **		√ (RNA-seq)		
5. (5)		√ (RNA-seq)		√ (RNA-seq)

* Studies which also indicated a subset of highly abundant miRNAs
GC-B: Germinal center B cells; NHL: non-Hodgkin lymphoma; RLNs: reactive lymph nodes; RT-qPCR: reverse transcription-quantitative polymerase chain reaction

Supplementary Table S2. Consensus human miRNA signature of cHL

miRNA	differentially expressed miRNAs									abundant miRNAs		
	HRS cells or cHL cell lines vs GC-B				cHL vs RLN	cHL vs others						
	Ref 3	Ref 3	Ref 4	Ref 5	Ref 1	Ref 2	Ref 2	Ref 2	Ref 5	Ref 2	Ref 4	Ref 5
	HRS cells	cHL cell lines	cHL cell lines	cHL cell lines	cHL cell lines vs RNL	CB-LCL cell lines	PMBL cell lines	BL cell lines	NHL cell lines	cHL cell lines	cHL cell lines	cHL cell lines
**hsa-miR-9-3p**			up									
**hsa-miR-9-5p**	up	up	up	up				up	up			
**hsa-miR-10b-5p**				up					up			
**hsa-miR-15b**						up		up		abundant		
**hsa-miR-16**	up	up				up		up		abundant		
**hsa-miR-17-5p**										abundant		
**hsa-miR-18a**	up	up										
**hsa-miR-19a**								up		abundant		
**hsa-miR-19b**						up				abundant		
**hsa-miR-20a**	up	up				up				abundant		
**hsa-miR-20b-5p**			down							abundant		
**hsa-miR-21-3p**				up					up			
**hsa-miR-21-5p**	up	up			up			up		abundant		abundant
**hsa-miR-23a-3p**			up	up				up	up			
**hsa-miR-23a-5p**			up	up					up			
**hsa-miR-23b-3p**				up	down				up			
**hsa-miR-23b-5p**				up					up			
**hsa-miR-24-2-5p**			up	up					up			
**hsa-miR-24-3p**	up							up				
**hsa-miR-25**		up				up		up		abundant		
**hsa-miR-26b**		up										
**hsa-miR-27a-3p**			up	up	up			up	up			
**hsa-miR-27a-5p**			up	up					up			
**hsa-miR-27b-5p**				up					up			
**hsa-miR-27b-3p**				up					up		abundant	
**hsa-miR-28-3p**			down									
**hsa-miR-28-5p**			down									
**hsa-miR-29a**								up		abundant		
**hsa-miR-29b**										abundant		
**hsa-miR-29c**								up		abundant		
**hsa-miR-30a-3p**			down									
**hsa-miR-30a-5p**	up	up	down	down					down			
**hsa-miR-30b**	up	up				up				abundant		
**hsa-miR-30e-5p**											abundant	
**hsa-miR-33**	down											
**hsa-miR-33b-5p**			up									
**hsa-miR-34a-5p**				up					up			
**has-miR-34b**						down						
**hsa-miR-92**						up		up				
**hsa-miR-92a-3p**										abundant	abundant	abundant
**hsa-miR-92b-3p**			up	up					up			
**hsa-miR-92b-5p**				up					up			
**hsa-miR-93**								up		abundant		
**hsa-miR-98-3p**			up	up					up			
**hsa-miR-98-5p**				up				up	up			
**hsa-miR-99a-5p**			up									
**hsa-miR-99b-3p**				up				up	up			
**has-miR100**						up						
**hsa-miR-101-5p**			up									
**hsa-miR-103**		up								abundant		
**hsa-miR-106a-5p**				down				up	down	abundant		
**hsa-miR-106b**								up		abundant		
**has-miR-107**							up					
**has-miR-1180-3p**				up					up			
**hsa-miR-125a**								up				
**hsa-miR-125b**	up											
**hsa-miR-126-3p**				up	down				up			
**hsa-miR-126-5p**				up					up			
**hsa-miR-129-5p**				up					up			
**hsa-miR-129-2-3p**				up					up			
**hsa-miR-130a-3p**			down									
**hsa-miR-130b**								up				
**hsa-miR-135a**					down		up					
**hsa-miR-135b**		up										
**hsa-miR-138-1-3p**			down									
**hsa-miR-138-5p**			down		down							

**has-miR-139**						down						
**hsa-miR-140**	up	up										
**hsa-miR-141-3p**			down									
**hsa-miR-142-3p**								up		abundant		
**hsa-miR-142-5p**										abundant	abundant	
**hsa-miR-143-3p**	down		down									
**hsa-miR-145**	up											
**hsa-miR-146b-3p**				up					up			
**hsa-miR-146b-5p**				up					up			
**hsa-miR-147**					up							
**hsa-miR-147b**			up									
**hsa-miR-148a-3p**			down	down					down			
**hsa-miR-148a-5p**			down	down					down			
**hsa-miR-149-5p**			up									
**hsa-miR-150-3p**			down	down					down			
**hsa-miR-150-5p**			down	down		down	down	down	down			
**hsa-miR-153-3p**			up			up		up				
**hsa-miR-155-3p**			up	up					up			
**hsa-miR-155-5p**	up	up	up	up				up	up	abundant	abundant	abundant
**hsa-miR-181a-2-3p**			down	down					down			
**hsa-miR-181a-5p**								down			abundant	
**hsa-miR-181c-3p**				up					up			
**hsa-miR-181d-5p**				up					up			
**hsa-miR-182**		down			up							
**hsa-miR-183**					up							
**hsa-miR-186**	up	up										
**hsa-miR-190a-5p**			up									
**hsa-miR-190**								up				
**hsa-miR-191-5p**								up		abundant	abundant	
**hsa-miR-195-3p**				up					up			
**hsa-miR-195-5p**			up	up					up			
**hsa-miR-196a-5p**	up	up	up	up					up			
**hsa-miR-196b-5p**				up					up			
**hsa-miR-200a**	down	down										
**hsa-miR-200b-3p**				down					down			
**hsa-miR-200c**	up											
**hsa-miR-203a-3p**	up											
**hsa-miR-204**					down							
**hsa-miR-205**					down							
**hsa-miR-206**						up						
**hsa-miR-208**								up				
**hsa-miR-214**	up											
**hsa-miR-216**					up							
**hsa-miR-218**								up				
**hsa-miR-223**	up											
**hsa-miR-223-3p**			down									
**hsa-miR-296-5p**			up									
**hsa-miR-301b-3p**			up	up					up			
**hsa-miR-320a**			up									
**hsa-miR-323**	up											
**hsa-miR-330-3p**			up	up					up			
**hsa-miR-330-5p**			up									
**hsa-miR-331**		up										
**hsa-miR-335**					down							
**hsa-miR-339-3p**				down					down			
**hsa-miR-340-5p**			down									
**hsa-miR-342-5p**				down		down			down			
**hsa-miR-345-5p**			up	up					up			
**hsa-miR-346**	up											
**hsa-miR-362**	down											
**hsa-miR-363-3p**	down		down									
**hsa-miR-373**	up											
**hsa-miR-374**	up	up										
**hsa-miR-375**	up											
**hsa-miR-376a**	down											
**hsa-miR-378a-3p**			up									
**hsa-miR-424-3p**			up	up					up			
**hsa-miR-424-5p**			up									
**hsa-miR-449a**				up					up			
**hsa-miR-449c-5p**				up					up			
**hsa-miR-450a-5p**			up	up					up			
**hsa-miR-450b-5p**			up	up					up			
**hsa-miR-455**	down	down										
**hsa-miR-459**		down										
**hsa-miR-485-3p**	up											
**hsa-miR-490**	up											
**hsa-miR-497-5p**			up	up					up			
**hsa-miR-503-5p**				up					up			
**hsa-miR-504-5p**				up					up			
**hsa-miR-520a#**	down	down										
**hsa-miR-520d#**		down										
**hsa-miR-526b**	up											
**hsa-miR-526c**		down										
**hsa-miR-539**	down											
**hsa-miR-542-3p**			up	up					up			
**hsa-miR-544**	up											
**hsa-miR-548h-5p**			down									
**hsa-miR-550a-3-5p**			up	up					up			
**hsa-miR-550a-3p**			up									
**hsa-miR-550a-5p**			up	up					up			
**hsa-miR-565**										abundant		
**hsa-miR-574**	down											
**hsa-miR-577**			down	down					down			
**hsa-miR-598-3p**			down	down					down			
**hsa-miR-609**	up											
**hsa-miR-610**	up											
**hsa-miR-614**	down	down										
**hsa-miR-615-3p**			up	up					up			

**hsa-miR-615-5p**				up					up			
**hsa-miR-625-5p**			up									
**hsa-miR-634**		down										
**hsa-miR-636**	up											
**hsa-miR-650**	down											
**hsa-miR-652**		down										
**hsa-miR-658**		down										
**hsa-miR-744-3p**			up									
**hsa-miR-760**			up									
**hsa-miR-766-5p**				down					down			
**hsa-miR-877-5p**			up									
**hsa-miR-888-5p**				up					up			
**hsa-miR-891a-5p**				up					up			
**hsa-miR-892a**				up					up			
**hsa-miR-944**			up									
**hsa-miR-1180-3p**				up					up			
**hsa-miR-1254**			up									
**hsa-miR-1273h-3p**				down					down			
**hsa-miR-1273h-5p**				down					down			
**hsa-miR-1276**			up									
**hsa-miR-1295a**				down					down			
**hsa-miR-1301-3p**			up	up					up			
**hsa-miR-2116-3p**			down									
**hsa-miR-3150b-3p**			down	down					down			
**hsa-miR-3158-3p**				down					down			
**hsa-miR-3176**			up									
**hsa-miR-3200-3p**			up									
**hsa-miR-3681-5p**			down									
**hsa-miR-3934-5p**				down					down			
**hsa-miR-4461**			up									
**hsa-miR-4488**				down					down			
**hsa-miR-4491**				down					down			
**hsa-miR-5100**			down									
**hsa-miR-5701**			up									
**hsa-miR-6500-3p**			down									
**hsa-miR-6842-3p**				down					down			
**hsa-miR-7705**			up									
**hsa-miR-7974**				down					down			
**hsa-let-7a-3p**			up							abundant		
**hsa-let-7a-5p**											abundant	abundant
**hsa-let-7b-3p**			up									
**hsa-let-7b-5p**			up									
**hsa-let-7e**						up		up				
**hsa-let-7f-5p**										abundant	abundant	abundant
**hsa-let-7f-2-3p**			up	up		up			up			
**hsa-let-7g**		up			up					abundant		

GC-B: Germinal center B cells; NHL: non-Hodgkin lymphoma; RLNs: reactive lymph nodes; CB-LCL: Germinal center B-cell-derived lymphoblastoid cell line; PMBL: primary mediastinal B-cell lymphoma; BL: Burkitt lymphoma

Supplementary Table S3. Pivotal cHL genes studied for experimentally validated miRNA regulation

Main theme	Sub theme	Selected pivotal gene
Loss of B cell phenotype	B-cell specific transcription factors	**PAX5, OCT2**, **PU.1, EBF1**, **ETS1**
Immune evasion	Antigen presentation	**HLA-A/B/C, B2M, TAP1, TAP2, CALR,** MICA, **MICB, HLA-II, CIITA, HLA-G**
	Immune checkpoint	**PD-L1**, PD-L2
	Immunosuppressive molecules	**Galectin-1, IL-10, TGF-beta**
	Chemokines shaping TME	CCL17, **CCL22**
Growth support signals	NF-KB pathway	**CD40,** CD30**, CD86, TNF-a, TNFAIP3, RELA, RELB, REL (CREL), NFKBIA (IκBα), NFKB1**
	Other pathways	**PDGFRA**, EPHB1. DDR2, TRKA, **c-MET, IGF1R, NOTCH1**, **JAG1, IL-21R, IL-6R**, **CSF1R, IL6,** IL7, **IL9, IL15**

Genes written in **bold** are experimentally validated miRNA targets (by Luciferase assay or Western Blot), details are shown in supplementary Table 4.

Supplementary Table S4. Overview of experimentally validated miRNA – target gene interactions sorted per main theme

4A. Loss of B-cell phenotype

Target gene	miRNA	Validated cell types	miRNA in cHL
PAX5 ↑	miR-338-3p	colon cancer (6)	
OCT2 ↓	miR-489-3p	renal cell carcinoma (7)	
	miR-630	renal cell carcinoma (7)	
PU.1 ↓	miR-150	autoimmune encephalitis (8)	down
	miR-155-5p*	acute myeloid leukemia (9)	up
EBF1 ↓	miR-19a-5p*	oral squamous cell carcinoma (10)	up
	miR-361-5p	adipocytes (11)	
	miR-574-5p	adipocytes (11)	down
	miR-98-5p	bladder cancer (12)	up
ETS-1 ↓	miR-9	gastric carcinoma (13)	up
	miR-145	gastric carcinoma (14)	up
	miR-155*	hematopoietic progenitor cells (15)	up
	miR-1	hepatocellular carcinoma (16); t helper 17 cells (17)	
	miR-499	hepatocellular carcinoma (16)	
	miR-200c	colorectal cancer (18)	up
	miR-222	metastatic melanoma and acute leukemia (19)	
	miR-125b	breast cancer (20)	up
	miR-144-3p	laryngeal squamous cell carcinoma (21)	
	miR-200b	endothelial cells (22)	down
	MiR-208	osteoblast cells (23)	up
	miR-221-3p	synovial fibroblasts (24)	

4B. Immune evasion

Target gene	miRNA	Validated cell types	miRNA in cHL
Antigen presentation			
HLA-A/B/C ↓	miR-744-5p	breast cancer; colon cancer (25)	
HLA-C ↓	miR-148a-3p	epstein-barr virus (ebv)-transformed b cell lines and t cell line (jurkat) (26)	down
B2M ↓	miR-335-5p	bladder cancer (27)	down
TAP1 ↓	miR-200a-5p	melanoma (28)	down
	miR-346	non-small cell lung cancer; cervical cancer (29)	up
TAP2 ↓	mIR-125a-5p	esophageal adenocarcinoma (30)	up
CALR ↓	miR-27a	colon cancer (31)	up
MICA ↓	-	-	-
MICB ↓	BART2-5p	colon cancer (32)	Expressed in cHL
CIITA ↓	miR-146b-5p	melanoma; cervical cancer ; gastric cancer; macrophages (33)	up
	let-7f-5p*	melanoma; cervical cancer ; gastric cancer; macrophages (33)	
	let-7i-5p	melanoma; cervical cancer ; gastric cancer; macrophages (33)	
	miR-185- 5p	melanoma; cervical cancer ; gastric cancer; macrophages (33)	
HLA-G ↑	miR-16-5p*	breast cancer; colon cancer (25)	up
	miR-148a-3p	choriocarcinoma (34); colon cancer (35);<br>oral squamous carcinoma (36);	down
	miR-744-5p	breast cancer; colon cancer (25)	
	miR-133a	trophoblast cell line (jeg-3) (37)	
	miR-148b	choriocarcinoma(34);	
	miR-152	choriocarcinoma (34); colon cancer (35);<br>human trophoblast cell line (jeg-3) (38);	
	miR-548q	renal cell carcinoma (39)	
	miR-628-5p	renal cell carcinoma (39)	
immune checkpoint			
PD-L1 ↑	miR-15b*	mesothelioma (40)	up
	miR-16-5p*	mesothelioma (40); gastric cancer (41); prostate cancer (42)	up
	miR-17-5p*	metastatic melanoma (43)	
	MiR-20b-5p*	colon cancer; lung cancer and breast cancer (44, 45)	down
	miR-34a	diffuse large b-cell lymphoma(46); non-small cell lung cancer (47); colon cancer (48); acute myeloid leukemia (49, 50)	up
	miR-34b	non-small cell lung cancer (47)	down
	miR-93-3p*	pancreatic cancer(51)	up
	miR-93-5p*	breast cancer (52)	up
	miR-106b-5p*	pancreatic cancer (51)	up
	miR-138-5p	colon cancer (53, 54)	down
	miR-140	osteosarcoma (55); non-small cell lung cancer (56); colon cancer (57); gastric cancer (58); cervical cancer (59)	up
	miR-142-5p*	cervical cancer (59); lung cancer (60); non-small cell lung cancer(61); pancreatic ductal adenocarcinoma (62); macrophages (63)	up
	miR-148a-3p	colon cancer (64), anaplastic thyroid carcinoma (65)	down
	miR-155-5p*	cervical cancer, lymphatic endothelial cells; dermal fibroblasts (66)	up
	miR-155*	b-cell lymphoma (67)	up

	miR-195-5p	diffuse large b-cell lymphoma (68, 69); gastric cancer (70); pancreatic cancer (71); prostate cancer (42); triple negative breast cancer (72)	up
	miR-200a	non-small cell lung cancer (73); breast cancer (74)	down
	miR-200b	non-small cell lung cancer (73); breast cancer (74); gastric cancer (75)	down
	miR-200c	non-small cell lung cancer (73); breast cancer (74); hbv+ human liver cell line (76); acute myeloid leukemia (49)	up
	miR-214-5p	bladder cancer (77)	up
	miR-340-5p	cervical cancer (59)	down
	miR-378a-3p	hepatocellular carcinoma (78)	up
	miR-424	ovarian cancer (79)	up
	miR-455-5p	hepatocellular carcinoma (80)	down
	miR-497	colon cancer (81); renal cell carcinoma (82); triple negative breast cancer (72)	up
	miR-766-5P	ovarian carcinoma (83)	down
	let-7* (no details about which let-7 member is used)	non-small cell lung cancer (84)	up
	miR-15a	mesothelioma(40)	
	miR-22	hematopoietic stem cells (85)	
	miR-105-5p	prostate cancer (85)	
	miR-152	gastric cancer (75)	
	miR-193a-3p	malignant pleural mesothelioma (40)	
	miR-383	cervical cancer (59)	
	miR-429	non-small cell lung cancer (86); gastric cancer (87)	
	miR-485-5p	non-small cell lung cancer (81)	
	miR-513	choanocytes; gastric cancer (88, 89)	
	miR-519d-3p	osteosarcoma (90)	
	miR-570	gastric cancer (89)	
	miR-576-3p	ovarian cancer (91)	
	miR-873	breast cancer (92)	
	miR-2467	epithelial ovarian carcinoma (93)	
	miR-3609	breast cancer (94)	
PD-L2 ↑	-	-	-
immunosuppressive molecules			
Galectin-1 ↑	miR-22	hepatocellular carcinoma (85)	
	miR-2467	epithelial ovarian carcinoma (93)	
IL10 ↑	miR-106a*	t cell line (jurkat)(95)	up/down
	miR-17-5p*	systemic lupus erythematosus (sle) (96)	
	miR-98-5p	ulcerative colitis (97)	up
	miR-374b-5p	deep venous thrombosis model (98)	up
	let-7c	t cell (99)	
	miR-194	b-cell lymphoma (100)	
TGF-beta ↑	miR-23	cardiac fibroblast (101)	up
	miR-24-3p	trabecular meshwork cells (tm) (102)	up
	miR-29b-3p*	trabecular meshwork cells (103, 104)	
	miR-130a-3p	autoimmune disease (105)	down
	miR-143-3p	mesangial cells (106)	down
	miR-744-5p	osteosarcoma, hepatocellular carcinoma, glioma (107-109)	up
	miR-124-3p	hypertrophic scar fibroblasts (110)	
	miR-211	immature osteoblasts (111)	
	miR-633	colon cancer (112)	
	miR-663a	lung cancer (113)	
	miR-515-5p	oral squamous cell carcinoma(114)	
chemokines shaping tme			
CCL17 ↑	-	-	-
CCL22 ↑	miR-23a	hepatocellular carcinoma (115)	up
	miR-34a	hepatocellular carcinoma (116)	up
	miR-30b*	lymphoma (117)	up
	miR-130a-5p	gastric cancer (118)	
	miR-9	cervical cancer (119)	up
	Let-7*	bone marrow-derived mesenchymal stem cells (120)	up
	miR-26a	hepatocellular carcinoma (121)	
	miR-223	tuberculosis (122)	up
	miR-217	ovarian cancer (123)	

4.3 Growth supporting signals

NF-KB pathway			
**Target gene**	**miRNA**	**Validated cell types**	**miRNA in cHL**
CD40 ↑	MiR-16*	myocarditis (124)	up
	miR-145	vascular smooth muscle cells (125)	up
	miR-146a	dendritic cell (126)	
	miR-424	umbilical vein endothelial cells (127)	up

	miR-503	umbilical vein endothelial cells (127)	up
	miR-1976	hek-293 cells (128)	
	MiR-195	non-alcoholic fatty liver disease (129)	up
	miR-590	endothelial cells (130)	
CD30 ↑	-	-	-
CD86 ↑	miR-146a	bone marrow-derived dcs (131)	
	miR-199a-3p	dendritic cells (132)	
TNF-α ↑	miR-19a*	esophageal squamous cell carcinoma (133)	up
	miR-140-5p	pulmonary artery smooth muscle cells (134)	up
	miR-125b	sepsis (135)	up
	miR-130a	sepsis (135)	down
TNFAIP3 ↓	miR-29c*	hepatocellular carcinoma (136)	up
	miR-125a	diffuse large b-cell lymphoma (137)	up
	miR-23a-3p	hodgkin lymphoma (5)	up
	MiR-27a-5p	hek-293 cells (137)	up
	miR-125b	diffuse large b-cell lymphoma (138)	up
RELA ↑	miR-27a-5p	hek-293 cells (138)	up
	miR-138	trophoblasts (139)	down
	miR-520	breast cancer (140)	down
	miR-373	breast cancer (140)	
	miR-155*	umbilical vein endothelial cells (141)	up
	miR-361	hepatocellular carcinoma (142)	
	miR-302b-3p	prostate cancer (143)	
	miR-124-3p	acute respiratory distress syndrome (144)	
	miR-7	gastric cancer (145)	
	miR-520c-3p	aortic vascular smooth muscle cells (141)	
RELB ↑	miR-146a	monocytes (146)	
REL (CREL)↑	miR-181a*	diffuse large b-cell lymphoma (147)	down
NFKBIA (IκBα)↓	miR-181a*	diffuse large b-cell lymphoma (147)	down
	miR-30e*	glioma (148)	
	miR-126	ulcerative colitis (149)	up
	miR-381a-3p	osteoarthritis (150)	
NFKB1 ↑	miR-181a*	diffuse large b-cell lymphoma (147)	down
	miR-508-3p	human gastric carcinoma(151)	
	miR-9	gastric cancer (152)	up
others			
PDGFRA ↑	miR-146a	leukemia (153)	
	miR-34a	pulmonary artery smooth muscle cells (154); lung cancer (155); glioma (156)	up
	miR-140-5p	ovarian cancer (157)	up
	miR-130b	fibroblasts (158)	up
	miR-491-5p	prostate cancer (159)	
	miR-218	fibroblasts (158)	up
	miR-34c	lung cancer (155)	
EPHB1	-	-	-
DDR2	-	-	-
TRKA	-	-	-
C-MET↑	miR-146a	colon cancer (160)	
	miR-206	nrf2-silenced cancer cells (161)	up
IGF1-R ↑	miR-30a-5p	melanoma (162)	up/down
	miR-29a-3p*	hepatocellular carcinoma (163)	up
	miR-145	bladder cancer (164)	up
	let-7*	primary spermatocytes (165)	up
	miR-16*	osteosarcoma (166)	up
	miR-15b*	glioblastoma cells (167)	up
	miR-143-3p	rheumatoid arthritis (168); squamous cell carcinoma (169)	down
	miR-195	non-small cell lung cancer (170)	up
	miR-100	nasopharyngeal carcinoma (171)	up
	miR-497	hepatocellular carcinoma (172)	up
	miR-99a	hepatocellular carcinoma (172); gastric cancer(173)	up
	miR-99b-5p	gastric cancer (174)	up
	miR-203a-3p	gastric cancer (174)	up
	miR-122	hepatocellular carcinoma (175); breast cancer(176)	
	miR-133b	adipose tissue-derived stem cells (177)	
	miR-223	cervical cancer (178)	up/down
	miR-383	glioma (179)	
	miR-181b	glioma (180)	
	miR-376a	melanoma (181)	down
	miR-376c	melanoma (181)	
	miR-486	lung cancer (182)	
NOTCH1 ↑	miR-34a	glioma (183); pancreatic cells (184)	up
	miR-582-5p	non-small cell lung cancer (185)	down
	miR-92a*	osteosarcoma (186)	up
	miR-363-3p	gastric cancer (187)	down
	miR-139-5p	colorectal cancer (188)	down

	miR-30	podocytes (189)	up/down
	miR-449a	hepatocellular carcinoma (190)	up
	miR-935	gastric signet ring cell carcinoma (191)	
	miR146b	cardiomyocytes (192)	up
	miR-34c	bone (193)	
	miR-181b	macrophages (194)	
JAG1 ↑	miR-21*	breast cancer (195); dendritic cells (196)	up
	miR-34a	prostate cancer (197); human epidermal keratinocytes (198); dendritic cells (196)	up
	miR-199b	ovarian cancer (199); hepatocellular carcinoma (200); porcine muscle satellite cells (201); ligamentum flavum cells (202)	
	miR-129-5p	non-small cell lung cancer (203)	up
	miR-539	wilms' tumor (204)	down
	miR-26b	nasopharyngeal carcinoma (205)	up
	MiR-224	pancreas neoplasm(206)	
	miR-506	osteosarcoma (207)	
	miR-524-5p	glioma (208)	
	miR-449a	skeletal muscle cells (209)	up
IL-21R ↑	miR-125a	gastric cancer (210)	up
	miR-30a	autoimmune encephalomyelitis (211)	up/down
IL-6R ↑	miR-449a	hepatitis(212)	up
CSF1R ↑	miR-34a	colorectal cancer (213); dendritic cells (214)	up
	miR-155*	dendritic cells (214)	up
	miR-22	dendritic cells (214)	
	miR-217	ovarian cancer (123)	
	let-7*	bone marrow-derived mesenchymal stem cells (120)	up
IL-6↑	miR-365	hek-293 cells (215)	
	miR-223	tuberculosis (122)	up
	miR-9	cervical cancer (119)	up
IL-7↑	-	-	-
IL-9 ↑	miR-208b-5p	non-small cell lung cancer (216)	down
IL-15 ↑	miR-15b*	myasthenia gravis(217)	up

*abundant miRNAs

References:

1. Navarro A, Gaya A, Martinez A, Urbano-Ispizua A, Pons A, Balagué O, et al. MicroRNA expression profiling in classic Hodgkin lymphoma. Blood. 2008;111:2825-32.
2. Gibcus JH, Tan LP, Harms G, Schakel RN, de Jong D, Blokzijl T, et al. Hodgkin lymphoma cell lines are characterized by a specific miRNA expression profile. Neoplasia (New York, NY). 2009;11:167-76.
3. Van Vlierberghe P, De Weer A, Mestdagh P, Feys T, De Preter K, De Paepe P, et al. Comparison of miRNA profiles of microdissected Hodgkin/Reed-Sternberg cells and Hodgkin cell lines versus CD77 + B-cells reveals a distinct subset of differentially expressed miRNAs. British Journal of Haematology. 2009;147:686-90.
4. Yuan Y, Kluiver J, Koerts J, de Jong D, Rutgers B, Abdul Razak FR, et al. miR-24-3p Is Overexpressed in Hodgkin Lymphoma and Protects Hodgkin and Reed-Sternberg Cells from Apoptosis. The American journal of pathology. 2017;187:1343-55.
5. Paczkowska J, Janiszewska J, Ustaszewski A, Bein J, Skalski M, Dzikiewicz-Krawczyk A, et al. Deregulated miRNAs Contribute to Silencing of B-Cell Specific Transcription Factors and Activation of NF-κB in Classical Hodgkin Lymphoma. Cancers (Basel). 2021;13(13).
6. Li W, He Y, Cheng Z. Long noncoding RNA XIST knockdown suppresses the growth of colorectal cancer cells via regulating microRNA-338-3p/PAX5 axis. European journal of cancer prevention : the official journal of the European Cancer Prevention Organisation (ECP). 2021;30(2):132-42.
7. Chen L, Chen L, Qin Z, Lei J, Ye S, Zeng K, et al. Upregulation of miR-489-3p and miR-630 inhibits oxaliplatin uptake in renal cell carcinoma by targeting OCT2. Acta Pharmaceutica Sinica B. 2019;9(5):1008-20.
8. Shakerian L, Ghorbani S, Talebi F, Noorbakhsh F. MicroRNA-150 targets PU.1 and regulates macrophage differentiation and function in experimental autoimmune encephalomyelitis. J Neuroimmunol. 2018;323:167-74.
9. Gerloff D, Grundler R, Wurm AA, Bräuer-Hartmann D, Katzerke C, Hartmann JU, et al. NF-κB/STAT5/miR-155 network targets PU.1 in FLT3-ITD-driven acute myeloid leukemia. Leukemia. 2015;29(3):535-47.
10. Liu G-M, Lu T-C, Sun M-L, Ji X, Zhao Y-A, Jia W-Y, et al. RP11-874J12.4 promotes oral squamous cell carcinoma tumorigenesis via the miR-19a-5p/EBF1 axis. J Oral Pathol Med. 2020;49(7):645-54.
11. Belarbi Y, Mejhert N, Gao H, Arner P, Rydén M, Kulyté A. MicroRNAs-361-5p and miR-574-5p associate with human adipose morphology and regulate EBF1 expression in white adipose tissue. Mol Cell Endocrinol. 2018;472:50-6.
12. Luo H, Yang L, Liu C, Wang X, Dong Q, Liu L, et al. TMPO-AS1/miR-98-5p/EBF1 feedback loop contributes to the progression of bladder cancer. The International Journal of Biochemistry & Cell Biology. 2020;122:105702.
13. Zheng L, Qi T, Yang D, Qi M, Li D, Xiang X, et al. microRNA-9 suppresses the proliferation, invasion and metastasis of gastric cancer cells through targeting cyclin D1 and Ets1. PLoS One. 2013;8(1):e55719.
14. Zheng L, Pu J, Qi T, Qi M, Li D, Xiang X, et al. miRNA-145 targets v-ets erythroblastosis virus E26 oncogene homolog 1 to suppress the invasion, metastasis, and angiogenesis of gastric cancer cells. Mol Cancer Res. 2013;11(2):182-93.
15. Romania P, Lulli V, Pelosi E, Biffoni M, Peschle C, Marziali G. MicroRNA 155 modulates megakaryopoiesis at progenitor and precursor level by targeting Ets-1 and Meis1 transcription factors. Br J Haematol. 2008;143(4):570-80.
16. Wei W, Hu Z, Fu H, Tie Y, Zhang H, Wu Y, et al. MicroRNA-1 and microRNA-499 downregulate the expression of the ets1 proto-oncogene in HepG2 cells. Oncol Rep. 2012;28(2):701-6.
17. Li L, Ma X, Zhao YF, Zhang C. MiR-1-3p facilitates Th17 differentiation associating with multiple sclerosis via targeting ETS1. Eur Rev Med Pharmacol Sci. 2020;24(12):6881-92.
18. Hur K, Toiyama Y, Takahashi M, Balaguer F, Nagasaka T, Koike J, et al. MicroRNA-200c modulates epithelial-to-mesenchymal transition (EMT) in human colorectal cancer metastasis. Gut. 2013;62(9):1315-26.
19. Coskun E, Neumann M, Schlee C, Liebertz F, Heesch S, Goekbuget N, et al. MicroRNA profiling reveals aberrant microRNA expression in adult ETP-ALL and functional studies implicate a role for miR-222 in acute leukemia. Leuk Res. 2013;37(6):647-56.
20. Zhang Y, Yan L-X, Wu Q-N, Du Z-M, Chen J, Liao D-Z, et al. miR-125b is methylated and functions as a tumor suppressor by regulating the ETS1 proto-oncogene in human invasive breast cancer. Cancer Res. 2011;71(10):3552-62.
21. Zhang S-Y, Lu Z-M, Lin Y-F, Chen L-S, Luo X-N, Song X-H, et al. miR-144-3p, a tumor suppressive microRNA targeting ETS-1 in laryngeal squamous cell carcinoma. Oncotarget. 2016;7(10):11637-50.
22. Chan YC, Khanna S, Roy S, Sen CK. miR-200b targets Ets-1 and is down-regulated by hypoxia to induce angiogenic response of endothelial cells. J Biol Chem. 2011;286(3):2047-56.
23. Itoh T, Takeda S, Akao Y. MicroRNA-208 modulates BMP-2-stimulated mouse preosteoblast differentiation by directly targeting V-ets erythroblastosis virus E26 oncogene homolog 1. J Biol Chem. 2010;285(36):27745-52.

24. Xu J, Liu Y, Deng M, Li J, Cai H, Meng Q, et al. MicroRNA221-3p modulates Ets-1 expression in synovial fibroblasts from patients with osteoarthritis of temporomandibular joint. Osteoarthritis Cartilage. 2016;24(11):2003-11.
25. Friedrich M, Vaxevanis CK, Biehl K, Mueller A, Seliger B. Targeting the coding sequence: opposing roles in regulating classical and non-classical MHC class I molecules by miR-16 and miR-744. Journal for immunotherapy of cancer. 2020;8.
26. Kulkarni S, Savan R, Qi Y, Gao X, Yuki Y, Bass SE, et al. Differential microRNA regulation of HLA-C expression and its association with HIV control. Nature. 2011;472(7344):495-8.
27. Sun M, Liu X, Zhao W, Zhang B, Deng P. Circ_0058063 contributes to cisplatin-resistance of bladder cancer cells by upregulating B2M through acting as RNA sponges for miR-335-5p. BMC Cancer. 2022;22(1):313.
28. Lazaridou MF, Gonschorek E, Massa C, Friedrich M, Handke D, Mueller A, et al. Identification of miR-200a-5p targeting the peptide transporter TAP1 and its association with the clinical outcome of melanoma patients. Oncoimmunology. 2020;9(1):1774323.
29. Bartoszewski R, Brewer JW, Rab A, Crossman DK, Bartoszewska S, Kapoor N, et al. The unfolded protein response (UPR)-activated transcription factor X-box-binding protein 1 (XBP1) induces microRNA-346 expression that targets the human antigen peptide transporter 1 (TAP1) mRNA and governs immune regulatory genes. J Biol Chem. 2011;286(48):41862-70.
30. Mari L, Hoefnagel SJM, Zito D, van de Meent M, van Endert P, Calpe S, et al. microRNA 125a Regulates MHC-I Expression on Esophageal Adenocarcinoma Cells, Associated With Suppression of Antitumor Immune Response and Poor Outcomes of Patients. Gastroenterology. 2018;155(3):784-98.
31. Colangelo T, Polcaro G, Ziccardi P, Pucci B, Muccillo L, Galgani M, et al. Proteomic screening identifies calreticulin as a MIR-27a direct target repressing MHC class I cell surface exposure in colorectal cancer. Cell Death and Disease. 2016;7:1-11.
32. Nachmani D, Stern-Ginossar N, Sarid R, Mandelboim O. Diverse herpesvirus microRNAs target the stress-induced immune ligand MICB to escape recognition by natural killer cells. Cell Host Microbe. 2009;5(4):376-85.
33. Codolo G, Toffoletto M, Chemello F, Coletta S, Soler Teixidor G, Battaggia G, et al. Helicobacter pylori Dampens HLA-II Expression on Macrophages via the Up-Regulation of miRNAs Targeting CIITA. Frontiers in Immunology. 2020;10.
34. Tan Z, Randall G, Fan J, Camoretti-Mercado B, Brockman-Schneider R, Pan L, et al. Allele-specific targeting of microRNAs to HLA-G and risk of asthma. Am J Hum Genet. 2007;81(4):829-34.
35. Manaster I, Goldman-Wohl D, Greenfield C, Nachmani D, Tsukerman P, Hamani Y, et al. MiRNA-mediated control of HLA-G expression and function. PLoS ONE. 2012;7:1-9.
36. Zhang Y, Jin X, Wang J. MiR-148a modulates the viability, migration and invasion of oral squamous cell carcinoma cells by regulating HLA-G expression. Molecular Medicine Reports. 2019;20:795-801.
37. Wang X, Li B, Wang J, Lei J, Liu C, Ma Y, et al. Evidence that miR-133a causes recurrent spontaneous abortion by reducing HLA-G expression. Reproductive BioMedicine Online. 2012;25:415-24.
38. Zhu X-m, Han T, Wang X-h, Li Y-h. BASIC SCIENCE : OBSTETRICS Overexpression of miR-152 leads to reduced expression of human leukocyte antigen-G and increased natural killer cell mediated cytolysis in JEG-3 cells. YMOB. 2010;202:592.e1-.e7.
39. Jasinski-Bergner S, Reches A, Stoehr C, Massa C, Gonschorek E, Huettelmaier S, et al. Identification of novel microRNAs regulating HLA-G expression and investigating their clinical relevance in renal cell carcinoma. Oncotarget. 2016;7(18):26866-78.
40. Kao SC, Cheng YY, Williams M, Kirschner MB, Madore J, Lum T, et al. Tumor Suppressor microRNAs Contribute to the Regulation of PD-L1 Expression in Malignant Pleural Mesothelioma. Journal of Thoracic Oncology. 2017;12(9):1421-33.
41. Li Z, Suo B, Long G, Gao Y, Song J, Zhang M, et al. Exosomal miRNA-16-5p Derived From M1 Macrophages Enhances T Cell-Dependent Immune Response by Regulating PD-L1 in Gastric Cancer. Front Cell Dev Biol. 2020;8:572689.
42. Tao Z, Xu S, Ruan H, Wang T, Song W, Qian L, et al. MiR-195/-16 Family Enhances Radiotherapy via T Cell Activation in the Tumor Microenvironment by Blocking the PD-L1 Immune Checkpoint. Cell Physiol Biochem. 2018;48(2):801-14.
43. Audrito V, Serra S, Stingi A, Orso F, Gaudino F, Bologna C, et al. PD-L1 up-regulation in melanoma increases disease aggressiveness and is mediated through miR-17-5p. Oncotarget. 2017;8(9):15894-911.
44. Xu YJ, Zhao JM, Ni XF, Wang W, Hu WW, Wu CP. LncRNA HCG18 suppresses CD8(+) T cells to confer resistance to cetuximab in colorectal cancer via miR-20b-5p/PD-L1 axis. Epigenomics. 2021;13(16):1281-97.
45. Jiang K, Zou H. microRNA-20b-5p overexpression combing Pembrolizumab potentiates cancer cells to radiation therapy via repressing programmed death-ligand 1. Bioengineered. 2022;13(1):917-29.

46. Anastasiadou E, Stroopinsky D, Alimperti S, Jiao AL, Pyzer AR, Cippitelli C, et al. Epstein-Barr virus-encoded EBNA2 alters immune checkpoint PD-L1 expression by downregulating miR-34a in B-cell lymphomas. Leukemia. 2019;33(1):132-47.
47. Cortez MA, Ivan C, Valdecanas D, Wang X, Peltier HJ, Ye Y, et al. PDL1 Regulation by p53 via miR-34. Journal of the National Cancer Institute. 2016;108:1-9.
48. Meng F, Chen Y, Yang M, Zhang H, Wang W. Concomitant inhibition of B7-H3 and PD-L1 expression by a novel and synthetic microRNA delivers potent antitumor activities in colorectal tumor models. Invest New Drugs. 2021;39(5):1267-74.
49. Pyzer AR, Stroopinsky D, Rosenblatt J, Anastasiadou E, Rajabi H, Washington A, et al. MUC1 inhibition leads to decrease in PD-L1 levels via upregulation of miRNAs. Leukemia. 2017;31(12):2780-90.
50. Wang X, Li J, Dong K, Lin F, Long M, Ouyang Y, et al. Tumor suppressor miR-34a targets PD-L1 and functions as a potential immunotherapeutic target in acute myeloid leukemia. Cellular Signalling. 2015;27:443-52.
51. Cioffi M, Trabulo SM, Vallespinos M, Raj D, Kheir TB, Lin M-L, et al. The miR-25-93-106b cluster regulates tumor metastasis and immune evasion via modulation of CXCL12 and PD-L1. Oncotarget. 2017;8(13):21609-25.
52. Yang M, Xiao R, Wang X, Xiong Y, Duan Z, Li D, et al. MiR-93-5p regulates tumorigenesis and tumor immunity by targeting PD-L1/CCND1 in breast cancer. Ann Transl Med. 2022;10(4):203.
53. Zhao L, Yu H, Yi S, Peng X, Su P, Xiao Z, et al. The tumor suppressor miR-138-5p targets PD-L1 in colorectal cancer. Oncotarget. 2016;7(29):45370-84.
54. Zhang XL, Xu LL, Wang F. Hsa_circ_0020397 regulates colorectal cancer cell viability, apoptosis and invasion by promoting the expression of the miR-138 targets TERT and PD-L1. Cell Biol Int. 2017;41(9):1056-64.
55. Ji X, Wang E, Tian F. MicroRNA-140 suppresses osteosarcoma tumor growth by enhancing anti-tumor immune response and blocking mTOR signaling. Biochem Biophys Res Commun. 2018;495(1):1342-8.
56. Xie WB, Liang LH, Wu KG, Wang LX, He X, Song C, et al. MiR-140 Expression Regulates Cell Proliferation and Targets PD-L1 in NSCLC. Cell Physiol Biochem. 2018;46(2):654-63.
57. Jiang W, Li T, Wang J, Jiao R, Shi X, Huang X, et al. miR-140-3p Suppresses Cell Growth And Induces Apoptosis In Colorectal Cancer By Targeting PD-L1. Onco Targets Ther. 2019;12:10275-85.
58. Zhao M, Liu Q, Liu W, Zhou H, Zang X, Lu J. MicroRNA-140 suppresses Helicobacter pylori-positive gastric cancer growth by enhancing the antitumor immune response. Mol Med Rep. 2019;20(3):2484-92.
59. Dong P, Xiong Y, Yu J, Chen L, Tao T, Yi S, et al. Control of PD-L1 expression by miR-140/142/340/383 and oncogenic activation of the OCT4-miR-18a pathway in cervical cancer. Oncogene. 2018;37(39):5257-68.
60. Jiang Y, Wang K, Lu X, Wang Y, Chen J. Cancer-associated fibroblasts-derived exosomes promote lung cancer progression by OIP5-AS1/ miR-142-5p/ PD-L1 axis. Mol Immunol. 2021;140:47-58.
61. Wan J, Ling X, Peng B, Ding G. miR-142-5p regulates CD4+ T cells in human non-small cell lung cancer through PD-L1 expression via the PTEN pathway. Oncol Rep. 2018;40(1):272-82.
62. Jia L, Xi Q, Wang H, Zhang Z, Liu H, Cheng Y, et al. miR-142-5p regulates tumor cell PD-L1 expression and enhances anti-tumor immunity. Biochemical and Biophysical Research Communications. 2017;488:425-31.
63. Zhen J, Chen W. MiR-142 inhibits cecal ligation and puncture (CLP)-induced inflammation via inhibiting PD-L1 expression in macrophages and improves survival in septic mice. Biomed Pharmacother. 2018;97:1479-85.
64. Ashizawa M, Okayama H, Ishigame T, Thar Min AK, Saito K, Ujiie D, et al. microRNA-148a-3p regulates immunosuppression in DNA mismatch repair-deficient colorectal cancer by targeting PD-L1. Molecular Cancer Research. 2019:molcanres.0831.2018.
65. Wang X, Zhang Y, Zheng J, Yao C, Lu X. LncRNA UCA1 attenuated the killing effect of cytotoxic CD8 + T cells on anaplastic thyroid carcinoma via miR-148a/PD-L1 pathway. Cancer Immunol Immunother. 2021;70(8):2235-45.
66. Yee D, Shah KM, Coles MC, Sharp TV, Lagos D. MicroRNA-155 induction via TNF-α and IFN-γ suppresses expression of programmed death ligand-1 (PD-L1) in human primary cells. J Biol Chem. 2017;292(50):20683-93.
67. Zheng Z, Sun R, Zhao HJ, Fu D, Zhong HJ, Weng XQ, et al. MiR155 sensitized B-lymphoma cells to anti-PD-L1 antibody via PD-1/PD-L1-mediated lymphoma cell interaction with CD8+T cells. Mol Cancer. 2019;18(1):54.
68. He B, Yan F, Wu C. Overexpressed miR-195 attenuated immune escape of diffuse large B-cell lymphoma by targeting PD-L1. Biomedicine & Pharmacotherapy. 2018;98:95-101.
69. Wang QM, Lian GY, Song Y, Huang YF, Gong Y. LncRNA MALAT1 promotes tumorigenesis and immune escape of diffuse large B cell lymphoma by sponging miR-195. Life Sci. 2019;231:116335.
70. Liu W, Chen H, Wang D. Protective role of astragaloside IV in gastric cancer through regulation of microRNA-195-5p-mediated PD-L1. Immunopharmacol Immunotoxicol. 2021;43(4):443-51.
71. Zhou WY, Zhang MM, Liu C, Kang Y, Wang JO, Yang XH. Long noncoding RNA LINC00473 drives the progression of pancreatic cancer via upregulating programmed death‐ligand 1 by sponging microRNA‐195‐5p. Journal of cellular physiology. 2019;234(12):23176-89.

72. Yang L, Cai Y, Zhang D, Sun J, Xu C, Zhao W, et al. miR-195/miR-497 Regulate CD274 Expression of Immune Regulatory Ligands in Triple-Negative Breast Cancer. J Breast Cancer. 2018;21(4):371-81.
73. Chen L, Gibbons DL, Goswami S, Cortez MA, Ahn YH, Byers LA, et al. Metastasis is regulated via microRNA-200/ZEB1 axis control of tumour cell PD-L1 expression and intratumoral immunosuppression. Nat Commun. 2014;5:5241.
74. Noman MZ, Janji B, Abdou A, Hasmim M, Terry S, Tan TZ, et al. The immune checkpoint ligand PD-L1 is upregulated in EMT-activated human breast cancer cells by a mechanism involving ZEB-1 and miR-200. Oncoimmunology. 2017;6(1):e1263412.
75. Xie G, Li W, Li R, Wu K, Zhao E, Zhang Y, et al. Helicobacter pylori promote B7-H1 expression by suppressing miR-152 and miR-200b in gastric cancer cells. PLoS ONE. 2017;12:1-13.
76. Sun C, Lan P, Han Q, Huang M, Zhang Z, Xu G, et al. Oncofetal gene SALL4 reactivation by hepatitis B virus counteracts MIR-200c in PD-L1-induced T cell exhaustion. Nature Communications. 2018;9.
77. Zhang M, Xu Y, Yin S, Qiu F. YY1-induced long non-coding RNA PSMA3 antisense RNA 1 functions as a competing endogenous RNA for microRNA 214-5p to expedite the viability and restrict the apoptosis of bladder cancer cells via regulating programmed cell death-ligand 1. Bioengineered. 2021;12(2):9150-61.
78. Li Y, Zhou T, Cheng X, Li D, Zhao M, Zheng WV. microRNA-378a-3p regulates the progression of hepatocellular carcinoma by regulating PD-L1 and STAT3. Bioengineered. 2022;13(3):4730-43.
79. Xu S, Tao Z, Hai B, Liang H, Shi Y, Wang T, et al. miR-424(322) reverses chemoresistance via T-cell immune response activation by blocking the PD-L1 immune checkpoint. Nat Commun. 2016;7:11406.
80. Zeng C, Ye S, Chen Y, Zhang Q, Luo Y, Gai L, et al. HOXA-AS3 Promotes Proliferation and Migration of Hepatocellular Carcinoma Cells via the miR-455-5p/PD-L1 Axis. J Immunol Res. 2021;2021:9289719.
81. Zhang N, Fan J, Deng Z. CircFOXK2 enhances tumorigenesis and immune evasion in non-small cell lung cancer by miR-485-5p/PD-L1 axis. Anticancer Drugs. 2022;33(5):437-47.
82. Qu F, Ye J, Pan X, Wang J, Gan S, Chu C, et al. MicroRNA-497-5p down-regulation increases PD-L1 expression in clear cell renal cell carcinoma. Journal of Drug Targeting. 2019;27:67-74.
83. Xu YJ, Zhao JM, Gao C, Ni XF, Wang W, Hu WW, et al. Hsa_circ_0136666 activates Treg-mediated immune escape of colorectal cancer via miR-497/PD-L1 pathway. Cell Signal. 2021;86:110095.
84. Li Y, Zhai Y, Chen Y. GATA1-induced upregulation of LINC01503 promotes carboplatin resistance in ovarian carcinoma by upregulating PD-L1 via sponging miR-766-5p. J Ovarian Res. 2021;14(1):108.
85. You Y, Tan JX, Dai HS, Chen HW, Xu XJ, Yang AG, et al. MiRNA-22 inhibits oncogene galectin-1 in hepatocellular carcinoma. Oncotarget. 2016;7(35):57099-116.
86. Wang R, Xuan Y, Zhao Y, Wang W, Ma P, Ju B, et al. Cationic Nanoparticulate System for Codelivery of MicroRNA-424 and Podophyllotoxin as a Multimodal Anticancer Therapy. Mol Pharm. 2022.
87. Mu L, Wang Y, Su H, Lin Y, Sui W, Yu X, et al. HIF1A-AS2 Promotes the Proliferation and Metastasis of Gastric Cancer Cells Through miR-429/PD-L1 Axis. Dig Dis Sci. 2021;66(12):4314-25.
88. Gong A-Y, Zhou R, Hu G, Li X, Splinter PL, O'Hara SP, et al. MicroRNA-513 regulates B7-H1 translation and is involved in IFN-gamma-induced B7-H1 expression in cholangiocytes. Journal of immunology (Baltimore, Md : 1950). 2009;182(3):1325-33.
89. Wang W, Sun J, Li F, Li R, Gu Y, Liu C, et al. A frequent somatic mutation in CD274 3'-UTR leads to protein over-expression in gastric cancer by disrupting miR-570 binding. Hum Mutat. 2012;33(3):480-4.
90. Wang J, Zhang Z, Qiu C. MicroRNA-519d-3p antagonizes osteosarcoma resistance against cisplatin by targeting PD-L1. Mol Carcinog. 2022;61(3):322-33.
91. Zuo Y, Zheng W, Tang Q, Liu J, Wang S, Xin C. miR-576-3p overexpression enhances cisplatin sensitivity of ovarian cancer cells by dysregulating PD-L1 and cyclin D1. Mol Med Rep. 2021;23(1).
92. Gao L, Guo Q, Li X, Yang X, Ni H, Wang T, et al. EBioMedicine MiR-873 / PD-L1 axis regulates the stemness of breast cancer cells. EBioMedicine. 2019:1-13.
93. Zhang PF, Wu J, Luo JH, Li KS, Wang F, Huang W, et al. SNHG22 overexpression indicates poor prognosis and induces chemotherapy resistance via the miR-2467/Gal-1 signaling pathway in epithelial ovarian carcinoma. Aging. 2019;11(19):8204-16.
94. Li D, Wang X, Yang M, Kan Q, Duan Z. miR3609 sensitizes breast cancer cells to adriamycin by blocking the programmed death-ligand 1 immune checkpoint. Exp Cell Res. 2019;380(1):20-8.
95. Sharma A, Kumar M, Aich J, Hariharan M, Brahmachari SK, Agrawal A, et al. Posttranscriptional regulation of interleukin-10 expression by hsa-miR-106a. Proceedings of the National Academy of Sciences of the United States of America. 2009;106(14):5761-6.
96. Xu L, Wang L, Shi Y, Deng Y, Oates JC, Kamen DL, et al. Up-Regulated Interleukin-10 Induced by E2F Transcription Factor 2-MicroRNA-17-5p Circuitry in Extrafollicular Effector B Cells Contributes to Autoantibody Production in Systemic Lupus Erythematosus. Arthritis Rheumatol. 2022;74(3):496-507.

97. Wang Y, Wang N, Cui L, Li Y, Cao Z, Wu X, et al. Long Non-coding RNA MEG3 Alleviated Ulcerative Colitis Through Upregulating miR-98-5p-Sponged IL-10. Inflammation. 2021;44(3):1049-59.
98. Zhang Y, Miao X, Zhang Z, Wei R, Sun S, Liang G, et al. miR-374b-5p is increased in deep vein thrombosis and negatively targets IL-10. J Mol Cell Cardiol. 2020;144:97-108.
99. Jiang L, Cheng Z, Qiu S, Que Z, Bao W, Jiang C, et al. Altered let-7 expression in Myasthenia gravis and let-7c mediated regulation of IL-10 by directly targeting IL-10 in Jurkat cells. Int Immunopharmacol. 2012;14(2):217-23.
100. Harris-Arnold A, Arnold CP, Schaffert S, Hatton O, Krams SM, Esquivel CO, et al. Epstein-Barr virus modulates host cell microRNA-194 to promote IL-10 production and B lymphoma cell survival. Am J Transplant. 2015;15(11):2814-24.
101. Yu RB, Li K, Wang G, Gao GM, Du JX. MiR-23 enhances cardiac fibroblast proliferation and suppresses fibroblast apoptosis via targeting TGF-β1 in atrial fibrillation. Eur Rev Med Pharmacol Sci. 2019;23(10):4419-24.
102. Luna C, Li G, Qiu J, Epstein DL, Gonzalez P. MicroRNA-24 regulates the processing of latent TGFβ1 during cyclic mechanical stress in human trabecular meshwork cells through direct targeting of FURIN. J Cell Physiol. 2011;226(5):1407-14.
103. Luna C, Li G, Qiu J, Epstein DL, Gonzalez P. Cross-talk between miR-29 and transforming growth factor-betas in trabecular meshwork cells. Invest Ophthalmol Vis Sci. 2011;52(6):3567-72.
104. Guo Y, Sun Z, Chen M, Lun J. LncRNA TUG1 Regulates Proliferation of Cardiac Fibroblast via the miR-29b-3p/TGF-β1 Axis. Front Cardiovasc Med. 2021;8:646806.
105. Zhao H, Li H, Du W, Zhang D, Ge J, Xue F, et al. Reduced MIR130A is involved in primary immune thrombocytopenia via targeting TGFB1 and IL18. Br J Haematol. 2014;166(5):767-73.
106. Xu J, Xiang P, Liu L, Sun J, Ye S. Metformin inhibits extracellular matrix accumulation, inflammation and proliferation of mesangial cells in diabetic nephropathy by regulating H19/miR-143-3p/TGF-β1 axis. J Pharm Pharmacol. 2020;72(8):1101-9.
107. Liang H, Li L, Zhu S, Tan J, Yang B, Wang X, et al. MicroRNA-744-5p suppresses tumorigenesis and metastasis of osteosarcoma through the p38 mitogen-activated protein kinases pathway by targeting transforming growth factor-beta 1. Bioengineered. 2022;13(5):12309-25.
108. Huang W, Chen Q, Dai J, Zhang Y, Yi Y, Wei X, et al. miR-744-5p suppresses tumor proliferation and metastasis by targeting transforming growth factor-beta 1 (TGF-β1) in hepatocellular carcinoma (HCC). J Gastrointest Oncol. 2021;12(4):1811-22.
109. Lou J, Wang P, Chang K, Wang G, Geng X, Wu Y, et al. Knocking down LINC01116 can inhibit the regulation of TGF-β through miR-774-5p axis and inhibit the occurrence and development of glioma. American journal of translational research. 2021;13(5):5702-19.
110. Zhang S, Pan S. miR-124-3p targeting of TGF-β1 inhibits the proliferation of hypertrophic scar fibroblasts. Adv Clin Exp Med. 2021;30(3):263-71.
111. Sun T, Yang D, Wu Y, Sheng Q. The function of microRNA-211 expression in post-fracture bone cell apoptosis involving the transforming growth factor-β/ phosphoinositide 3-kinase signaling pathway. J Int Med Res. 2020;48(7):300060520926353-.
112. Li P, Song J, Du H, Lu Y, Dong S, Zhou S, et al. MicroRNA-663 prevents monocrotaline-induced pulmonary arterial hypertension by targeting TGF-β1/smad2/3 signaling. J Mol Cell Cardiol. 2021;161:9-22.
113. Mody HR, Hung SW, AlSaggar M, Griffin J, Govindarajan R. Inhibition of S-Adenosylmethionine-Dependent Methyltransferase Attenuates TGFβ1-Induced EMT and Metastasis in Pancreatic Cancer: Putative Roles of miR-663a and miR-4787-5p. Mol Cancer Res. 2016;14(11):1124-35.
114. Yan J, Xu H. Regulation of transforming growth factor-beta1 by circANKS1B/miR-515-5p affects the metastatic potential and cisplatin resistance in oral squamous cell carcinoma. Bioengineered. 2021;12(2):12420-30.
115. Li Z-Q, Wang H-Y, Zeng Q-L, Yan J-Y, Hu Y-S, Li H, et al. p65/miR-23a/CCL22 axis regulated regulatory T cells recruitment in hepatitis B virus positive hepatocellular carcinoma. Cancer Med. 2020;9(2):711-23.
116. Yang P, Li Q-J, Feng Y, Zhang Y, Markowitz GJ, Ning S, et al. TGF-β-miR-34a-CCL22 signaling-induced Treg cell recruitment promotes venous metastases of HBV-positive hepatocellular carcinoma. Cancer Cell. 2012;22(3):291-303.
117. Wang J-H, Liu F, Duan X-H, Hao C-X, Liu X-X, Lu Y-J, et al. [MiR-30b Regulates the Cisplatin-Resistance of Human NK/T Cell Lypnphoma Cell Lines SNK-6 and YTS by Targeting the CCL22]. Zhongguo Shi Yan Xue Ye Xue Za Zhi. 2019;27(6):1838-44.
118. Fang Q-L, Li K-C, Wang L, Gu X-L, Song R-J, Lu S. Targeted Inhibition of CCL22 by miR-130a-5p Can Enhance the Sensitivity of Cisplatin-Resistant Gastric Cancer Cells to Chemotherapy. Cancer Manag Res. 2020;12:3865-75.
119. Zhang J, Jia J, Zhao L, Li X, Xie Q, Chen X, et al. Down-regulation of microRNA-9 leads to activation of IL-6/Jak/STAT3 pathway through directly targeting IL-6 in HeLa cell. Mol Carcinog. 2016;55(5):732-42.

120. Sung S-Y, Liao C-H, Wu H-P, Hsiao W-C, Wu IH, Jinpu, et al. Loss of let-7 microRNA upregulates IL-6 in bone marrow-derived mesenchymal stem cells triggering a reactive stromal response to prostate cancer. PLoS One. 2013;8(8):e71637.
121. Yang X, Liang L, Zhang X-F, Jia H-L, Qin Y, Zhu X-C, et al. MicroRNA-26a suppresses tumor growth and metastasis of human hepatocellular carcinoma by targeting interleukin-6-Stat3 pathway. Hepatology. 2013;58(1):158-70.
122. Dorhoi A, Iannaccone M, Farinacci M, Faé KC, Schreiber J, Moura-Alves P, et al. MicroRNA-223 controls susceptibility to tuberculosis by regulating lung neutrophil recruitment. J Clin Invest. 2013;123(11):4836-48.
123. Jiang B, Zhu S-J, Xiao S-S, Xue M. MiR-217 Inhibits M2-Like Macrophage Polarization by Suppressing Secretion of Interleukin-6 in Ovarian Cancer. Inflammation. 2019;42(5):1517-29.
124. Li Q-Q, Xi J, Li B-Q, Li N. MiR-16, as a potential NF-κB-related miRNA, exerts anti-inflammatory effects on LPS-induced myocarditis via mediating CD40 expression: A preliminary study. J Biochem Mol Toxicol. 2020;34(2):e22426.
125. Guo X, Li D, Chen M, Chen L, Zhang B, Wu T, et al. miRNA-145 inhibits VSMC proliferation by targeting CD40. Scientific Reports. 2016;6(1):35302.
126. Chen T, Li Z, Tu j, Zhu W, Ge J, Zheng X, et al. MicroRNA-146a regulates the maturation process and pro-inflammatory cytokine secretion by targeting CD40L in oxLDL-stimulated dendritic cells. FEBS Letters. 2011;585(3):567-73.
127. Lee A, Papangeli I, Park Y, Jeong H-N, Choi J, Kang H, et al. A PPARγ-dependent miR-424/503-CD40 axis regulates inflammation mediated angiogenesis. Sci Rep. 2017;7(1):2528.
128. Garcia Lacarte M, Alfredo M, Zulet M, Milagro F. Implication of miR-612 and miR-1976 in the regulation of TP53 and CD40 and their relationship in the response to specific weight-loss diets. PLOS ONE. 2018;13:e0201217.
129. Li Y, Jiang H-T, Han L-B, Xiao L, Gan J-H. MiR-195 regulates CD40 to maintain Th17/Treg balance in rats with non-alcoholic fatty liver disease. Biomedicine & Pharmacotherapy. 2020;124:109930.
130. Wu T, Xiang Y, Lv Y, Li D, Yu L, Guo R. miR-590-3p mediates the protective effect of curcumin on injured endothelial cells induced by angiotensin II. Am J Transl Res. 2017;9(2):289-300.
131. Tang H, Lai Y, Zheng J, Chen K, Jiang H, Xu G. MiR-146a Promotes Tolerogenic Properties of Dendritic Cells and through Targeting Notch1 Signaling. Immunol Invest. 2020;49(5):555-70.
132. Xiong A, Wang J, Mao XL, Jiang Y, Fan Y. MiR‑199a‑3p modulates the function of dendritic cells involved in transplantation tolerance by targeting CD86. HLA. 2019;94(6):493-503.
133. Liu M, Wang Z, Yang S, Zhang W, He S, Hu C, et al. TNF-α is a novel target of miR-19a. International journal of oncology. 2011;38(4):1013-22.
134. Zhu TT, Zhang WF, Yin YL, Liu YH, Song P, Xu J, et al. MicroRNA-140-5p targeting tumor necrosis factor-α prevents pulmonary arterial hypertension. Journal of cellular physiology. 2019;234(6):9535-50.
135. Guan Y, Yao H, Wang J, Sun K, Cao L, Wang Y. NF-κB-DICER-miRs Axis Regulates TNF-α Expression in Responses to Endotoxin Stress. International journal of biological sciences. 2015;11(11):1257-68.
136. Wang CM, Wang Y, Fan CG, Xu FF, Sun WS, Liu YG, et al. miR-29c targets TNFAIP3, inhibits cell proliferation and induces apoptosis in hepatitis B virus-related hepatocellular carcinoma. Biochemical and biophysical research communications. 2011;411(3):586-92.
137. Kim SW, Ramasamy K, Bouamar H, Lin AP, Jiang D, Aguiar RC. MicroRNAs miR-125a and miR-125b constitutively activate the NF-κB pathway by targeting the tumor necrosis factor alpha-induced protein 3 (TNFAIP3, A20). Proceedings of the National Academy of Sciences of the United States of America. 2012;109(20):7865-70.
138. Romay MC, Che N, Becker SN, Pouldar D, Hagopian R, Xiao X, et al. Regulation of NF-κB signaling by oxidized glycerophospholipid and IL-1β induced miRs-21-3p and -27a-5p in human aortic endothelial cells. Journal of lipid research. 2015;56(1):38-50.
139. Yu G, Mu H, Zhou H, Fang F, Cui Y, Wu Q, et al. MicroRNA-361 suppresses the biological processes of hepatic stellate cells in HBV-relative hepatic fibrosis by NF-kappaB p65. Cells & development. 2021;167:203711.
140. Yin A, Chen Q, Zhong M, Jia B. MicroRNA-138 improves LPS-induced trophoblast dysfunction through targeting RELA and NF-κB signaling. Cell Cycle. 2021;20(5-6):508-21.
141. Wu XY, Fan WD, Fang R, Wu GF. Regulation of microRNA-155 in endothelial inflammation by targeting nuclear factor (NF)-κB P65. Journal of cellular biochemistry. 2014;115(11):1928-36.
142. Liang Y, Xie J, Che D, Zhang C, Lin Y, Feng L, et al. MiR-124-3p helps to protect against acute respiratory distress syndrome by targeting p65. Bioscience reports. 2020;40(5).
143. Cao H, Wang D, Sun P, Chen L, Feng Y, Gao R. RNA-seq reveals microRNA-302b as a suppressor of prostate cancer epithelial-mesenchymal transition by targeting RELA/NF-κB. American journal of cancer research. 2021;11(11):5715-25.
144. Wang J, Hu X, Hu X, Gao F, Li M, Cui Y, et al. MicroRNA-520c-3p targeting of RelA/p65 suppresses atherosclerotic plaque formation. Int J Biochem Cell Biol. 2021;131:105873.

145. Jin HF, Wang JF, Shao M, Zhou K, Ma X, Lv XP. Down-Regulation of miR-7 in Gastric Cancer Is Associated With Elevated LDH-A Expression and Chemoresistance to Cisplatin. Front Cell Dev Biol. 2020;8:555937.
146. Etzrodt M, Cortez-Retamozo V, Newton A, Zhao J, Ng A, Wildgruber M, et al. Regulation of monocyte functional heterogeneity by miR-146a and Relb. Cell reports. 2012;1(4):317-24.
147. Kozloski GA, Jiang X, Bhatt S, Ruiz J, Vega F, Shaknovich R, et al. miR-181a negatively regulates NF-κB signaling and affects activated B-cell-like diffuse large B-cell lymphoma pathogenesis. Blood. 2016;127(23):2856-66.
148. Jiang L, Lin C, Song L, Wu J, Chen B, Ying Z, et al. MicroRNA-30e* promotes human glioma cell invasiveness in an orthotopic xenotransplantation model by disrupting the NF-κB/IκBα negative feedback loop. J Clin Invest. 2012;122(1):33-47.
149. Feng X, Wang H, Ye S, Guan J, Tan W, Cheng S, et al. Up-regulation of microRNA-126 may contribute to pathogenesis of ulcerative colitis via regulating NF-kappaB inhibitor IκBα. PLoS One. 2012;7(12):e52782.
150. Xia S, Yan K, Wang Y. Increased miR-381a-3p Contributes to Osteoarthritis by Targeting IkBα. Ann Clin Lab Sci. 2016;46(3):247-53.
151. Huang T, Kang W, Zhang B, Wu F, Dong Y, Tong JH, et al. miR-508-3p concordantly silences NFKB1 and RELA to inactivate canonical NF-κB signaling in gastric carcinogenesis. Mol Cancer. 2016;15:9.
152. Bahrami A, Jafari A, Ferns GA. The dual role of microRNA-9 in gastrointestinal cancers: oncomiR or tumor suppressor? Biomed Pharmacother. 2022;145:112394.
153. Li L, Zhu S, Li Y, Cao W, Qiao X. Downregulation of miR-146a promotes proliferation and migration of AOB-treated embryoid body via PDGFRA induction. Mol Med Rep. 2018;17(2):2586-92.
154. Wang P, Xu J, Hou Z, Wang F, Song Y, Wang J, et al. miRNA-34a promotes proliferation of human pulmonary artery smooth muscle cells by targeting PDGFRA. Cell Prolif. 2016;49(4):484-93.
155. Garofalo M, Jeon Y-J, Nuovo GJ, Middleton J, Secchiero P, Joshi P, et al. Correction: MiR-34a/c-Dependent PDGFR-α/β Downregulation Inhibits Tumorigenesis and Enhances TRAIL-Induced Apoptosis in Lung Cancer. PLoS One. 2022;17(4):e0267628.
156. Silber J, Jacobsen A, Ozawa T, Harinath G, Pedraza A, Sander C, et al. miR-34a repression in proneural malignant gliomas upregulates expression of its target PDGFRA and promotes tumorigenesis. PLoS One. 2012;7(3):e33844.
157. Lan H, Chen W, He G, Yang S. miR-140-5p inhibits ovarian cancer growth partially by repression of PDGFRA. Biomed Pharmacother. 2015;75:117-22.
158. Bobbs AS, Saarela AV, Yatskievych TA, Antin PB. Fibroblast Growth Factor (FGF) Signaling during Gastrulation Negatively Modulates the Abundance of MicroRNAs That Regulate Proteins Required for Cell Migration and Embryo Patterning. Journal of Biological Chemistry. 2012;287(46):38505-14.
159. Xu Y, Hou R, Lu Q, Zhang Y, Chen L, Zheng Y, et al. MiR-491-5p negatively regulates cell proliferation and motility by targeting PDGFRA in prostate cancer. Am J Cancer Res. 2017;7(12):2545-53.
160. Bleau A-M, Redrado M, Nistal-Villan E, Villalba M, Exposito F, Redin E, et al. miR-146a targets c-met and abolishes colorectal cancer liver metastasis. Cancer Lett. 2018;414:257-67.
161. Choi B-H, Ryu DY, Ryoo I-G, Kwak M-K. NFE2L2/NRF2 silencing-inducible miR-206 targets c-MET/EGFR and suppresses BCRP/ABCG2 in cancer cells. Oncotarget. 2017;8(63):107188-205.
162. Li Y, Zhang J, Liu Y, Zhang B, Zhong F, Wang S, et al. MiR-30a-5p confers cisplatin resistance by regulating IGF1R expression in melanoma cells. BMC Cancer. 2018;18(1):404.
163. Wang X, Liu S, Cao L, Zhang T, Yue D, Wang L, et al. miR-29a-3p suppresses cell proliferation and migration by downregulating IGF1R in hepatocellular carcinoma. Oncotarget. 2017;8(49):86592-603.
164. Zhu Z, Xu T, Wang L, Wang X, Zhong S, Xu C, et al. MicroRNA-145 directly targets the insulin-like growth factor receptor I in human bladder cancer cells. FEBS Lett. 2014;588(17):3180-5.
165. Shen G, Wu R, Liu B, Dong W, Tu Z, Yang J, et al. Upstream and downstream mechanisms for the promoting effects of IGF-1 on differentiation of spermatogonia to primary spermatocytes. Life Sci. 2014;101(1-2):49-55.
166. Chen L, Wang Q, Wang G-D, Wang H-S, Huang Y, Liu X-M, et al. miR-16 inhibits cell proliferation by targeting IGF1R and the Raf1-MEK1/2-ERK1/2 pathway in osteosarcoma. FEBS Lett. 2013;587(9):1366-72.
167. Wang J, Liu H, Tian L, Wang F, Han L, Zhang W, et al. miR-15b Inhibits the Progression of Glioblastoma Cells Through Targeting Insulin-like Growth Factor Receptor 1. Horm Cancer. 2017;8(1):49-57.
168. Yang Z, Wang J, Pan Z, Zhang Y. miR-143-3p regulates cell proliferation and apoptosis by targeting IGF1R and IGFBP5 and regulating the Ras/p38 MAPK signaling pathway in rheumatoid arthritis. Exp Ther Med. 2018;15(4):3781-90.
169. Qian Y, Teng Y, Li Y, Lin X, Guan M, Li Y, et al. MiR-143-3p suppresses the progression of nasal squamous cell carcinoma by targeting Bcl-2 and IGF1R. Biochem Biophys Res Commun. 2019;518(3):492-9.
170. Wang X, Wang Y, Lan H, Li J. MiR-195 inhibits the growth and metastasis of NSCLC cells by targeting IGF1R. Tumour Biol. 2014;35(9):8765-70.

171. Sun X, Liu X, Wang Y, Yang S, Chen Y, Yuan T. miR-100 inhibits the migration and invasion of nasopharyngeal carcinoma by targeting IGF1R. Oncol Lett. 2018;15(6):8333-8.
172. Cheng H, Xue J, Yang S, Chen Y, Wang Y, Zhu Y, et al. Co-targeting of IGF1R/mTOR pathway by miR-497 and miR-99a impairs hepatocellular carcinoma development. Oncotarget. 2017;8(29):47984-97.
173. Xu XL, Guo AX, Pan QY, Chang AL, Zhao CR. MiR-99a suppresses cell migration and invasion by regulating IGF1R in gastric cancer. Eur Rev Med Pharmacol Sci. 2019;23(17):7375-82.
174. Wang Z, Zhao Z, Yang Y, Luo M, Zhang M, Wang X, et al. MiR-99b-5p and miR-203a-3p Function as Tumor Suppressors by Targeting IGF-1R in Gastric Cancer. Sci Rep. 2018;8(1):10119.
175. Bai S, Nasser MW, Wang B, Hsu S-H, Datta J, Kutay H, et al. MicroRNA-122 inhibits tumorigenic properties of hepatocellular carcinoma cells and sensitizes these cells to sorafenib. J Biol Chem. 2009;284(46):32015-27.
176. Wang B, Wang H, Yang Z. MiR-122 inhibits cell proliferation and tumorigenesis of breast cancer by targeting IGF1R. PLoS One. 2012;7(10):e47053.
177. Ning H, Huang Y-C, Banie L, Hung S, Lin G, Li L-C, et al. MicroRNA regulation of neuron-like differentiation of adipose tissue-derived stem cells. Differentiation. 2009;78(5):253-9.
178. Jia CY, Li HH, Zhu XC, Dong YW, Fu D, Zhao QL, et al. MiR-223 suppresses cell proliferation by targeting IGF-1R. PLoS One. 2011;6(11):e27008.
179. He Z, Cen D, Luo X, Li D, Li P, Liang L, et al. Downregulation of miR-383 promotes glioma cell invasion by targeting insulin-like growth factor 1 receptor. Med Oncol. 2013;30(2):557.
180. Shi Z-M, Wang X-F, Qian X, Tao T, Wang L, Chen Q-D, et al. MiRNA-181b suppresses IGF-1R and functions as a tumor suppressor gene in gliomas. RNA. 2013;19(4):552-60.
181. Zehavi L, Avraham R, Barzilai A, Bar-Ilan D, Navon R, Sidi Y, et al. Silencing of a large microRNA cluster on human chromosome 14q32 in melanoma: biological effects of mir-376a and mir-376c on insulin growth factor 1 receptor. Mol Cancer. 2012;11:44.
182. Peng Y, Dai Y, Hitchcock C, Yang X, Kassis ES, Liu L, et al. Insulin growth factor signaling is regulated by microRNA-486, an underexpressed microRNA in lung cancer. Proc Natl Acad Sci U S A. 2013;110(37):15043-8.
183. Xu H, Zhang Y, Qi L, Ding L, Jiang H, Yu H. NFIX Circular RNA Promotes Glioma Progression by Regulating miR-34a-5p via Notch Signaling Pathway. Front Mol Neurosci. 2018;11:225.
184. Li Y, Zhang T, Zhou Y, Sun Y, Cao Y, Chang X, et al. A Presenilin/Notch1 pathway regulated by miR-375, miR-30a, and miR-34a mediates glucotoxicity induced-pancreatic beta cell apoptosis. Sci Rep. 2016;6:36136.
185. Liu J, Liu S, Deng X, Rao J, Huang K, Xu G, et al. MicroRNA-582-5p suppresses non-small cell lung cancer cells growth and invasion via downregulating NOTCH1. PLoS One. 2019;14(6):e0217652.
186. Liu Q, Song Y, Duan X, Chang Y, Guo J. MiR-92a Inhibits the Progress of Osteosarcoma Cells and Increases the Cisplatin Sensitivity by Targeting Notch1. Biomed Res Int. 2018;2018:9870693.
187. Song B, Yan J, Liu C, Zhou H, Zheng Y. Tumor Suppressor Role of miR-363-3p in Gastric Cancer. Med Sci Monit. 2015;21:4074-80.
188. Song M, Yin Y, Zhang J, Zhang B, Bian Z, Quan C, et al. Correction to: MiR-139-5p inhibits migration and invasion of colorectal cancer by downregulating AMFR and NOTCH1. Protein Cell. 2021;12(8):668-70.
189. Wu J, Zheng C, Fan Y, Zeng C, Chen Z, Qin W, et al. Downregulation of microRNA-30 facilitates podocyte injury and is prevented by glucocorticoids. J Am Soc Nephrol. 2014;25(1):92-104.
190. Han B, Huang J, Yang Z, Zhang J, Wang X, Xu N, et al. miR-449a Is Related to Short-Term Recurrence of Hepatocellular Carcinoma and Inhibits Migration and Invasion by Targeting Notch1. Onco Targets Ther. 2019;12:10975-87.
191. Yan C, Yu J, Kang W, Liu Y, Ma Z, Zhou L. miR-935 suppresses gastric signet ring cell carcinoma tumorigenesis by targeting Notch1 expression. Biochem Biophys Res Commun. 2016;470(1):68-74.
192. Wang X, Yu Y. MiR-146b protect against sepsis induced mice myocardial injury through inhibition of Notch1. J Mol Histol. 2018;49(4):411-7.
193. Bae Y, Yang T, Zeng H-C, Campeau PM, Chen Y, Bertin T, et al. miRNA-34c regulates Notch signaling during bone development. Hum Mol Genet. 2012;21(13):2991-3000.
194. An T-H, He Q-W, Xia Y-P, Chen S-C, Baral S, Mao L, et al. MiR-181b Antagonizes Atherosclerotic Plaque Vulnerability Through Modulating Macrophage Polarization by Directly Targeting Notch1. Mol Neurobiol. 2017;54(8):6329-41.
195. Selcuklu SD, Donoghue MTA, Kerin MJ, Spillane C. Regulatory interplay between miR-21, JAG1 and 17beta-estradiol (E2) in breast cancer cells. Biochem Biophys Res Commun. 2012;423(2):234-9.
196. Hashimi ST, Fulcher JA, Chang MH, Gov L, Wang S, Lee B. MicroRNA profiling identifies miR-34a and miR-21 and their target genes JAG1 and WNT1 in the coordinate regulation of dendritic cell differentiation. Blood. 2009;114(2):404-14.

197. Liu X, Luo X, Wu Y, Xia D, Chen W, Fang Z, et al. MicroRNA-34a Attenuates Paclitaxel Resistance in Prostate Cancer Cells via Direct Suppression of JAG1/Notch1 Axis. Cell Physiol Biochem. 2018;50(1):261-76.
198. Gao Y, Yang M, Wei L, Liang X, Wu F, Huang Y, et al. miR-34a-5p Inhibits Cell Proliferation, Migration and Invasion Through Targeting JAG1/Notch1 Pathway in HPV-Infected Human Epidermal Keratinocytes. Pathol Oncol Res. 2020;26(3):1851-9.
199. Liu MX, Siu MKY, Liu SS, Yam JWP, Ngan HYS, Chan DW. Epigenetic silencing of microRNA-199b-5p is associated with acquired chemoresistance via activation of JAG1-Notch1 signaling in ovarian cancer. Oncotarget. 2014;5(4):944-58.
200. Li GL, Yuan JH, Zhuang GD, Wu DQ. miR-199b exerts tumor suppressive functions in hepatocellular carcinoma by directly targeting JAG1. Eur Rev Med Pharmacol Sci. 2018;22(22):7679-87.
201. Zhu L, Hou L, Ou J, Xu G, Jiang F, Hu C, et al. MiR-199b represses porcine muscle satellite cells proliferation by targeting JAG1. Gene. 2019;691:24-33.
202. Qu X, Chen Z, Fan D, Sun C, Zeng Y, Guo Z, et al. MiR-199b-5p inhibits osteogenic differentiation in ligamentum flavum cells by targeting JAG1 and modulating the Notch signalling pathway. J Cell Mol Med. 2017;21(6):1159-70.
203. Wang Y, Zhang L, Yang J, Sun R. LncRNA KCNQ1OT1 promotes cell proliferation, migration and invasion via regulating miR-129-5p/JAG1 axis in non-small cell lung cancer. Cancer Cell Int. 2020;20:144.
204. Su H, Wang X, Song J, Wang Y, Zhao Y, Meng J. MicroRNA-539 inhibits the progression of Wilms' Tumor through downregulation of JAG1 and Notch1/3. Cancer Biomark. 2019;24(1):125-33.
205. Shi L, Yin W, Zhang Z, Shi G. Down-regulation of miR-26b induces cisplatin resistance in nasopharyngeal carcinoma by repressing JAG1. FEBS Open Bio. 2016;6(12):1211-9.
206. Zhang B, Guo X, Zhang J, Liu X, Zhan X, Li Z. MicroRNA-224 is downregulated in mucinous cystic neoplasms of the pancreas and may regulate tumorigenesis by targeting Jagged1. Molecular Medicine Reports. 2014;10(6):3303-9.
207. Hu M, Yuan X, Liu Y, Tang S, Miao J, Zhou Q, et al. IL-1β-induced NF-κB activation down-regulates miR-506 expression to promotes osteosarcoma cell growth through JAG1. Biomed Pharmacother. 2017;95:1147-55.
208. Chen L, Zhang W, Yan W, Han L, Zhang K, Shi Z, et al. The putative tumor suppressor miR-524-5p directly targets Jagged-1 and Hes-1 in glioma. Carcinogenesis. 2012;33(11):2276-82.
209. Poddar S, Kesharwani D, Datta M. miR-449a regulates insulin signalling by targeting the Notch ligand, Jag1 in skeletal muscle cells. Cell Commun Signal. 2019;17(1):84.
210. Yan L, Zhang J, Guo D, Ma J, Shui S-F, Han X-W. IL-21R functions as an oncogenic factor and is regulated by the lncRNA MALAT1/miR-125a-3p axis in gastric cancer. Int J Oncol. 2019;54(1):7-16.
211. Qu X, Zhou J, Wang T, Han J, Ma L, Yu H, et al. MiR-30a inhibits Th17 differentiation and demyelination of EAE mice by targeting the IL-21R. Brain, Behavior, and Immunity. 2016;57:193-9.
212. Sarma NJ, Tiriveedhi V, Crippin JS, Chapman WC, Mohanakumar T. Hepatitis C virus-induced changes in microRNA 107 (miRNA-107) and miRNA-449a modulate CCL2 by targeting the interleukin-6 receptor complex in hepatitis. J Virol. 2014;88(7):3733-43.
213. Shi X, Kaller M, Rokavec M, Kirchner T, Horst D, Hermeking H. Characterization of a p53/miR-34a/CSF1R/STAT3 Feedback Loop in Colorectal Cancer. Cell Mol Gastroenterol Hepatol. 2020;10(2):391-418.
214. Riepsaame J, van Oudenaren A, den Broeder BJH, van Ijcken WFJ, Pothof J, Leenen PJM. MicroRNA-Mediated Down-Regulation of M-CSF Receptor Contributes to Maturation of Mouse Monocyte-Derived Dendritic Cells. Front Immunol. 2013;4:353.
215. Xu Z, Xiao S-B, Xu P, Xie Q, Cao L, Wang D, et al. miR-365, a Novel Negative Regulator of Interleukin-6 Gene Expression, Is Cooperatively Regulated by Sp1 and NF-κB. Journal of Biological Chemistry. 2011;286(24):21401-12.
216. Ma J, Tong Hf, Lin Jh, Chen Fn, Wu Cx, Cao Cz, et al. miR-208b-5p inhibits invasion of non-small cell lung cancer through the STAT3 pathway by targeting interleukin-9. Oncology Letters. 2020;20(1):299-307.
217. Shi L, Liu T, Zhang M, Guo Y, Song C, Song D, et al. miR-15b is Downregulated in Myasthenia Gravis Patients and Directly Regulates the Expression of Interleukin-15 (IL-15) in Experimental Myasthenia Gravis Mice. Med Sci Monit. 2015;21:1774-80.
